# Cardiac Surgery and Blood-Saving Techniques: An Update

**DOI:** 10.7759/cureus.21222

**Published:** 2022-01-13

**Authors:** Muhammad Saad Yousuf, Khalid Samad, Syed Shabbir Ahmed, Khalid M Siddiqui, Hameed Ullah

**Affiliations:** 1 Anaesthesiology, The Aga Khan University Hospital, Karachi, PAK; 2 Anaesthesia and Critical Care, The Aga Khan University Hospital, Karachi, PAK; 3 Anaesthesia and Critical Care, Aga Khan Health Service, Pakistan (AKHS,P), Karachi, PAK

**Keywords:** blood transfusion, cardiopulmonary bypass, blood loss, blood conservative strategies, cardiac surgery

## Abstract

Cardiac surgery is typically attributed with a significant risk of intraoperative blood loss and allogeneic blood transfusions. Intraoperative blood loss, allogenic blood transfusions, high dose anticoagulation requirement, and interactions with cardiopulmonary bypass (CPB) have all been linked to cardiac surgeries. To reduce unnecessary transfusions and their negative effects, it is recommended to follow evidence-based multidisciplinary strategies, which are collectively termed patient blood management (PBM). This review highlights the most recent blood conservation strategies in adult cardiac surgery, which can be employed pre-operatively, intra-operatively, and postoperatively, to enhance red cell mass and attenuate the utilization of packed red blood cells (PRBCs) and other blood products.

## Introduction and background

Historically, allogeneic blood transfusion (ABT) has been considered the only possible treatment for bleeding in major surgical operations [1`]. Possible transfusion-related adverse effects are infections, immunosuppressant effects, allergic reactions, and potential death [1]. Furthermore, the availability and increased cost to preserve blood are associated with organizational and economic problems.

Cardiac surgeries are associated with intraoperative blood loss, ABT, high dose anticoagulation requirement, and interactions with cardiopulmonary bypass (CPB). Patients undergoing cardiac surgery account for 15-20% of all perioperative ABTs [2,3]. To reduce unnecessary transfusions and their negative effects, it is recommended to follow evidence-based multidisciplinary strategies, which are collectively termed patient blood management (PBM). PBM entails maintenance of an adequate hemoglobin level, improves hemostasis, minimizes blood loss, and allows for transfusion of allogeneic blood only when required [4]. Recently, the European Association for Cardio-Thoracic Surgery (EACTS) and the European Association of Cardiothoracic Anaesthesiology (EACTA) recommended an evidence-based guideline for PBM in adult cardiac surgery as given in Table 1 [5].

**Table 1:** Recommendations for patient blood management in adult cardiac surgery AT: antithrombin; ACT: activated clotting time; CPB: cardiopulmonary bypass; CABG: coronary artery bypass grafting; DDAVP: 1-deamino-8-D-arginine vasopressin (desmopressin); FFP: fresh frozen plasma; MUF: modified ultrafiltration; PRBC: packed red blood cells; rFVIIa: recombinant factor VIIa; RAP: retrograde autologous priming

Recommended	Should Be Considered	Not Recommended
Limitation of hemodilution	Continue Aspirin in CABG	Preoperative erythrocyte transfusion in anaemic patients
Routine use of antifibrinolytics	Cell salvage, MUF, and RAP should be implemented	AT supplementation to minimize bleeding following CPB
Transfusion of PRBC based on the patient clinical condition rather than a haemoglobin level	Consider Heparin level measurements over ACT-guided heparin management	Prophylactic Fibrinogen, FFP, DDAVP or rFVIIa administration
PRBCs of all ages	Protamine to heparin dosing ratio <1:1	
Multidisciplinary team approach		

The purpose of this article is to review the most recent blood conservation strategies in adult cardiac surgery, which can be employed preoperatively, intra-operatively, and postoperatively, to enhance red cell mass and attenuate the utilization of packed red blood cells (PRBCs) and other blood products.

## Review

Blood conservation and transfusion strategies

Transfusion trigger is a term used to describe the conditions under which transfusions are reasonable and for which no further justification is needed [6]. A hemoglobin level of 9-10 gm/dl enhances capillary perfusion, reduces viscosity, and helps to increase the oxygenation of tissues. In surgical patients without any risk of ischemia, a hemoglobin level of 7 gm/dl is considered to be an appropriate transfusion threshold, whereas a 10 gm/dl threshold can be rationalized for patients with limited cardio-respiratory functions [7]. Blood transfusion decisions should include clinical judgment, as most clinical practitioners have transfused PRBCs by conducting risk or benefit assessments on the basis of their own experience and interpretation of evidence-based guidelines for individual patients. Compared to standard transfusion practices, a transfusion algorithm based on thromboelastographic parameters results in reduced requirements for transfusion and blood loss in both routine and high-risk cardiac procedures and is, therefore, better than treatment based solely on clinical experience and blood loss estimates [8].

Preoperative strategies

Preoperative Anemia Management

The incidence of preoperative anemia of any cause in cardiac surgery is approximately 40% [9].One small prospective study identified iron deficiency anemia as the most common form of preoperative anemia [10]. Oral iron requires at least six weeks to improve the hemoglobin level and may be ineffective [11]. Intravenous iron has been shown to be effective and safe [12]. The Prophylaxis for Thromboembolism in Critical Care Trial (PROTECT) showed that perioperative iron isomaltoside (1000mg) had a positive effect on postoperative Hb levels [13], whereas transfusion rates were comparable in both groups.

A meta-analysis of preoperative erythropoietin (EPO) administration in cardiac surgical patients showed that it was associated with a substantial reduction in the risk of ABT exposure with or without preoperative autologous donation (PAD) [14]. The warning alerts issued associated with the use of EPO as issued by the FDA, namely thrombotic events and promotion of tumor growth, should be carefully considered when weighing the risks and benefits of such therapies [15]. The guidelines recommend using a brief course of EPO with iron in patients having increased risk of postoperative anemia and in those who refuse transfusion (e.g., Jehovah's Witness) (Class IIa, Level of Evidence B) [16]. PAD candidates may also benefit from EPO (Class IIb, Level of Evidence A). The best PBM practices, thus, include diagnosis and management of preoperative anemia, reducing the requirement of ABT, and postponing elective surgery until the anemia has been corrected [11].

Autologous Transfusion

Compared with the allogeneic donated blood, the use of autologous blood is a potentially appealing and less harmful technique. It includes a preoperative autologous donation, perioperative acute normovolemic hemodilution (ANH), and intraoperative or postoperative cell salvage.

Preoperative Autologous Donation (PAD)

This technique involves repeated blood donation of approximately 450 ml, which begins about five weeks preceding surgery. The last donation should take place at least 48-72 hours before the operation to equilibrate the blood volume. Clearly labeled citrated phosphate dextrose blood bags are required to collect the blood, which is then conventionally stored in the blood bank. PAD minimizes the risks of viral transmission, immune-mediated hemolysis, and febrile or allergic reactions. There is no immune modulation, and this can reduce the risks of infection after surgery as well as recurrence of cancer [17].

The main drawbacks are increased logistic requirements and preparation well in advance of surgery, repeated phlebotomy prior to surgery, and benefits reserved only for elective cases. Up to 50% of pre-donated blood is unused; this waste results in a higher cost per unit of blood compared to allogeneic blood, along with the cost of administering PAD programs [8]. Iron therapy and erythropoietin can be used to increase the concentration of hemoglobin; this, however, contributes to the cost and is not without negative consequences and is therefore not usually recommended [18]. The PAD contraindications include unstable angina and severe aortic stenosis, with the exclusion of a significant percentage of cardiac surgical patients (those with anemia, cyanotic heart disease, ischemic heart disease, or uncontrolled hypertension); hence, despite its recommendations [19], the involvement of PAD in heart surgery is still under consideration. However, patients with uncommon blood types or having cross-matching difficulties, or where there is insufficient blood supply, are scenarios where PAD may still have a place [20].

Intraoperative strategies

Acute Normovolaemic Hemodilution (ANH)

ANH is carried out shortly after the induction of anesthesia in the anesthetic room. A wide-bore IV cannula is placed to remove a certain amount (approximately 15 to 20 ml/kg) of whole blood from the patient before heparinization and CPB, which will be labeled and placed in the operating room during the procedure. Crystalloid or colloid is used to restore blood volume. The whole blood collected is usually transfused back to the patient after protamine reversal. The same exclusion criteria are applied to this technique as to the PAD. The use of ANH reduces the requirements for ABT by preserving RBCs, coagulation factors, and platelets, and by improving perfusion during CPB by decreasing blood viscosity [21]. Barile et al. [22] carried out a meta-analysis of the use of ANH and found significant reductions in the number of allogeneic RBC units used and the amount of bleeding.

ANH should be used in patients with high preoperative Hb levels who can tolerate large amounts of blood removal and are at risk of major blood loss during surgery [23]. Reduction in ABT occurs most notably in >800 mL of ANH phlebotomy. In a multicenter, observational study [24], researchers found that the number of ABTs was reduced in all ANH volumes. With enhanced ANH volume removal, this reduction could become more pronounced. They also noted lower rates of fresh frozen plasma (FFP) and platelet transfusion in those with ANH, as well as lower acute kidney injury and lower rates of 30-day mortality and length of hospital stay. Zhou et al. [25]. found that even those undergoing low-volume ANH (5-8 mL/kg) required significantly fewer RBC units on an intraoperative basis. This reduction did not result in a reduction in FFP or platelet transfusions, postoperative or total perioperative allogeneic transfusions, or a significant difference in postoperative outcomes. Therefore, ANH is not routinely practiced unless a patient's Hb concentration is elevated (>15 gm/dl), enabling a higher volume of blood to be removed [23]. Thus, in combination with other methods of blood conservation, its use is limited in selected patients and could be used as part of a multipronged strategy (Class IIb, Level of Evidence A) [16].

Pharmacological Therapy

By acting on hemostasis, coagulation, and/or fibrinolysis, pharmacological therapies help to reduce blood loss intraoperatively and include desmopressin acetate, aprotinin, lysine analogs, and recombinant activated factor VII (rFVIIa).

Desmopressin

Desmopressin (DDAVP) is a synthetic vasopressin analogue that has been developed to prevent and treat hemorrhage in patients having mild hemophilia A and type 1 von Willebrand deficiency. DDAVP stimulates the endothelial cells to release the von Willebrand factor. A systematic Cochrane review showed that DDAVP prophylactic use did not reduce the possibility of blood loss or transfusions [26,27]. In addition, its effect may be stronger in certain subgroups, such as patients taking antiplatelet drugs, those with platelet dysfunction, and those who have been exposed to CPB for a long time [26]. In short, DDAVP is not recommended for prophylactic use; however, it may be beneficial in patients having impaired platelet functions or bleeding disorders [28].

Antifibrinolytics

These are commonly used in cardiac surgery to minimize bleeding, blood transfusions, and reoperations due to bleeding. They consist of the use of three agents: aprotinin, tranexamic acid (TXA), and ɛ-aminocaproic acid (EACA). The randomized control trial for Aspirin and Tranexamic Acid for Coronary Artery Surgery (ATACAS) has shown a reduced risk of reoperation and the need for any blood products using tranexamic acid [29]. On average, patients receiving either TXA or EACA experienced a decrease in blood transfusion, but the use of EACA did not minimize the incidence of re-exploration of bleeding compared to TXA [30] and was related to a higher risk of renal injury [31]. Neither drug has been shown to increase thrombotic events, but high doses of TXA are known to increase the risk of seizures [29].

Aprotinin has been extensively studied in patients who have undergone cardiac surgery. This reduces the rate of allogeneic transfusions by >30% during the perioperative period. Concerns have been raised, however, about possible serious risks associated with the use of this medicine: increased incidence of renal failure, myocardial infarction, stroke, and mortality [32]. A higher mortality rate was associated with aprotinin than those who received TXA or EACA, according to the Blood Conservation Using Antifibrinolytics (BART) study [33]. Following affirmation of the substantial fundamental limits of the BART trial [34,35], the European Medicines Agency lifted marketing restrictions in Canada and Europe in 2012 [36,37]. As a result, during isolated CABG surgery, aprotinin was confined to patients who were at greater risk of significant blood loss.

Antifibrinolytic drugs are therefore advised to be used during cardiac procedures (Class I, Level of Evidence A) [16] but the optimal dosage, efficacy, and safety profile studies should be designed to observe the adverse effects and their impact on bleeding.

Recombinant Factor VIIa (rFVIIa)

Pharmacological concentrations of rFVIIa may increase the formation of thrombin on activated platelets accrued at the vascular damage site. In cardiac surgery, the non-label need of rFVIIa [38] has increased primarily for the management of refractory bleeding, both during and after surgery. rFVIIa appears to be a promising treatment for uncontrollable bleeding caused by complex coagulopathies as a result of trauma or surgery. Thromboembolic events are common complications (deep vein thrombosis, pulmonary embolus, stroke, etc.) associated with the use of rFVIIa. A large retrospective study has shown that the incidence of thrombotic events occurs across different dosing regimens, in which cerebrovascular accidents were the most frequently occurring adverse event at a dose of 81-100µg⁄kg [39]. rFVIIa should not be used prophylactically in cardiac surgery; instead, it should be used therapeutically only in patients with non-surgical bleeding who do not respond to standard hemostatic treatment following CPB procedures. (Class IIb Evidence level B) [16].

Methods used by perfusionists

Minimally Invasive Extracorporeal Circulation Circuits (MiECC)

Shorter artificial tubing lengths, small priming volumes (600-750ml), a smaller artificial surface without venous reservoir, a heparin-coated biocompatible tubing, and centrifugal pumps are common features of MiECC. To prevent blood-air contact, systems are completely closed and involve the separation of bloodshed. Substantial fluctuations in flow due to venous return problems, because of the lack of open reservoir, as well as increased dependence on vasopressors to sustain pressure, are some of the drawbacks of using this method [40].

According to a systematic review and meta-analysis, the use of MiECC resulted in a significant reduction in the risk of transfusion but no difference in the rate of reoperation for persistent bleeding [41,42]. The Minimal Invasive Extra-Corporal Technologies International Society (MiECTiS) recommends that MiECC reduces hemodilution, postoperative bleeding, and the need for RBC transfusion while improving hematocrit (Class I, Level of Evidence A), particularly in patients who are at risk of hemodilution (small adults) or who refuse ABT [40].

Viscoelastic Testing

This evaluates whole blood coagulation, platelet function, and fibrinolytic activity. Early use of thromboelastography helps to diagnose the specific cause of coagulopathic bleeding, make decisions about the transfusion of FFP, platelets, and cryoprecipitate, and prevent unwarranted blood products. According to Deppe et al [43], the point-of-care (POC) tests minimized not only the exposure of ABT, RBC, FFP, and platelet transfusion, but also the risk of re-exploration of the chest. There is a lack of sufficient evidence of the use of POC platelet function tests to assess platelet dysfunction caused by antiplatelet agents and CPB [44]. Platelet dysfunction caused by CPB can be detected by post-bypass POC tests, which can also predict postoperative bleeding [45]. POC tests are therefore strongly recommended to guide the strategy of transfusion [23].

Retrograde Autologous Priming (RAP)

The use of the patient's own venous blood to prime the CPB circuit through retrograde flow can reduce hemodilution, resulting in less anemia and transfusion requirements. It is generally avoided in patients having severe aortic stenosis, because it is associated with hypotension and the use of vasopressors. According to the largest meta-analysis, RAP was related to a substantial decline in the number of intraoperative and perioperative patients exposed to ABT, as well as a decline in the number of blood transfusion units [46]. As a result, RAP is used as part of a blood conservation strategy to avoid transfusions and is only given to patients with low hemoglobin mass [23].

Cell Salvage

The process of collecting, filtering, and washing blood from the surgical field to produce autologous blood for transfusion back to the patient is known as cell salvage. Red cells are maintained but plasma, platelets, heparin, free hemoglobin, and inflammatory mediators are discarded along with a washing solution. This method can be used intraoperatively or postoperatively. Modern machines extract blood, which is then combined with heparinized normal saline or citrate anticoagulant. Blood can be given back to the patient after cardiac surgery by using cell-saving devices or from the cardiotomy suction via the bypass circuit. Direct re-infusion during periods of rapid hemorrhage may be lifesaving but unwashed blood contains lipid microemboli, gaseous emboli, and non-cellular debris, which can cause organ dysfunction, especially in the kidneys and brain [47]. Cell salvage has become a standard of care in cardiac surgery. Compared with banked RBCs, salvaged blood has normal levels of 2, 3-DPG, no left shift in the hemoglobin-oxygen saturation curve, and better cell membrane deformability [48].

In a meta-analysis, Wang et al [49] found that cell salvage reduced the ABT exposure rate while having no effect on the number of transfused FFP or platelet patients. Similarly, a Cochrane review in 2010 found that the risk of ABT was reduced by an average of 34% in cardiac surgical patients who received cell salvage [50]. Based on the meta-analysis [49], the 2014 European Society of Cardiology (ESC)/EACTS guidelines on myocardial revascularization advocate using cell salvage [51]. Red cell salvage is also recommended by the Society of Thoracic Surgeons (STS) and the Society of Cardiovascular Anesthesiologists (SCA) as a routine procedure. (Class I, Level of Evidence A) [16].

Ultrafiltration

Ultrafiltration avoids hemodilution by eliminating water and low molecular weight molecules from the CPB circuit, resulting in protein-rich enriched whole blood that can be delivered back to the patient. Three variants are used in cardiac surgery: (1) modified ultrafiltration (MUF) (2) zero balance ultrafiltration (ZBUF) and (3) conventional ultrafiltration.

MUF: In this method, excess fluid from the remaining blood is discarded after the termination of the CPB. It is likely to be underused in adults, but it has become the standard of care for pediatric congenital cardiac surgery. Luciani et al [52]. found that the proportion of patients who did not receive any blood products with MUF was higher, in addition to lowering the mean volume of RBCs transfused for each patient. Zahoor and colleagues [53] noticed that the MUF-treated group had significantly less blood loss and needed less PRBC, FFP, and platelet transfusions after 24 hours. Thus, it is recommended as a blood conservation strategy in adults undergoing cardiac surgery with CPB [16].

ZBUF:This is similar to conventional ultrafiltration, but it replaces the lost volume with a balanced electrolyte solution. This technique of ultrafiltration occurs during CPB. The main theoretical benefit is that ZBUF can be used to reduce the number of inflammatory mediators released when a foreign surface comes into contact with blood [54].In patients who received ZBUF versus those who did not, there was no significant difference in ICU length of stay, ventilation duration, chest tube output, or other parameters [55]. Therefore, for blood conservation, the advantages of using ZBUF are not well defined [16].

Postoperative strategies

Cell Salvage (Postoperative)

The infection rates, mortality rates, and the length of hospital stays have all decreased because of postoperative cell salvage [56]. Unwashed salvaged blood from postoperative surgical drains includes an elevated amount of inflammatory mediators, fibrin split products, interleukins, fat emboli, and complement factors, which could lead to inflammatory complications [57]. Patients receiving postoperative cell salvage blood had a 15.6% lower risk of allogeneic blood exposure during cardiac surgery [58], but it may compromise coagulation when large amounts of cell salvaged blood (> 1000 ml) are retransfused [59]. Thus, it is recommended to process the mediastinal shed blood by centrifugation and reinfuse it (Class IIb, Level of Evidence B) [16]. Therefore, cell salvage should be considered on a regular basis to avoid transfusions.

Avoiding Iatrogenic Blood Loss

In critically ill patients, diagnostic phlebotomy is the major source of iatrogenic blood loss. The complex cardiac surgical procedures were associated with the greater phlebotomy volume and increased ABT [60]. It has also been shown that the use of 1 mL of blood gas syringe instead of 3 mL of syringe reduced the amount of blood collected by 60% without affecting the integrity of the sample [61]. Since samples of less than 0.5 mL of blood are often required, the POC test is also an appealing substitute to standard laboratory tests [62]. Therefore, it is recommended to implement various strategies to avoid iatrogenic blood loss [23].

## Conclusions

In cardiac surgery, the employment of blood conservation strategies that include aggressive use of PAD, low CPB prime, and effective RAP, as well as traditional cell saver approaches, is beneficial. Permissive anemia is not harmful and does not increase the risk of complications. Avoiding blood transfusions may help to lower the risk of postoperative complications and long-term mortality. Clinical outcome studies are uncommon and sometimes underpowered, making it difficult to draw accurate results, particularly in adult patients. Randomized trials comparing the present standard practices of blood transfusion in cardiac surgery to a comprehensive blood conservation program are more important than ever.
